# Synthesis, Structure, Spectra, and Applications of Metal-Organic Frameworks: Basolite C-300

**DOI:** 10.3390/ijms26125777

**Published:** 2025-06-16

**Authors:** Gabriela Camarillo-Martínez, Evelia Martínez-Cano, Abraham Zepeda-Navarro, Jorge Luis Guzmán-Mar, Egla Yareth Bivián-Castro

**Affiliations:** 1 Centro Universitario de los Lagos, Universidad de Guadalajara, Av. Enrique Díaz de León 1144, Col. Paseos de la Montaña, Lagos de Moreno 47460, Jalisco, Mexico; gabriela.cmartinez@academicos.udg.mx (G.C.-M.); evelia.martinez@academicos.udg.mx (E.M.-C.); abraham.zepeda4162@alumnos.udg.mx (A.Z.-N.); 2 Facultad de Ciencias Químicas, Universidad Autonoma de Nuevo León (UANL), Ave. Universidad s/n, Cd. Universitaria, San Nicolas de los Garza 66455, Nuevo León, Mexico

**Keywords:** MOFs, HKUST-1, Basolite C-300, copper Metal-organic frameworks, coordination polymers

## Abstract

Metal-organic frameworks or MOFs are coordination polymers consisting of cationic metal centers liked by ligands. These coordination polymers have repeating entities that extend in one, two, or three dimensions through various Metal-ligand covalent bonds. The structural diversity of MOFs allows for the fine-tuning of properties like pore size, stability, and functionality, making them ideal for a wide range of industrial, environmental, and biomedical applications. Basolite C-300, HKUST-1 or [Cu_3_(btc)_2_(H_2_O)_3_], is one of the most studied three-dimensional porous frameworks. It is a commercially available MOF, easily produced under laboratory conditions. Its unique cubic structure, with multiple pore and adsorption sites, enhances its properties. This article reviews the conventional, new, and non-conventional methods of MOF and Basolite C-300 synthesis. In addition, the structural and spectral characterization of Basolite C-300 and its analogues is described, using spectroscopic and complementary multi-techniques to obtain fundamental knowledge about their structure. Finally, the applications of Basolite C-300 and similar MOFs are discussed, emphasizing their importance in industry and materials, technologies aimed at addressing global environmental and energy-related challenges, and biomedical applications.

## 1. Introduction

Metal-organic frameworks (MOFs) are hybrid materials that combine inorganic and organic components, characterized by their porous, crystalline, and network-like structures. These frameworks are constructed through the coordination of metal ions or clusters with organic ligands. The specific coordination geometries of the metals and ligands influence their diverse periodic arrangements and porosity levels [1]. A variety of metals and ligands can be employed to design MOFs with tailored properties, making them suitable for applications such as industrial use (gas separation, storage, and adsorption), biomedical (drug delivery, bioimaging, phototherapy), and environmental applications (catalysis). HKUST-1 (Hong Kong University of Science and Technology) is one of the most studied three-dimensional porous frameworks, alongside IRMOF-1. The structure of this material is described as [Cu_3_(btc)_2_(H_2_O)_3_] and is also now known by other names such as Cu-btc and MOF-199 and is marketed as Basolite C-300 by BASF Industries. It was developed by Chui and collaborators in 1999 [2,3]. It consists of a dinuclear copper (II) unit, Cu_2_(CO_2_)_4_, where each metal ion is coordinated to four oxygen atoms of the carboxylate groups of the 1,3,5-benzenetricarboxylic acid (btc) ligand in a paddlewheel arrangement similar to other copper compounds [4]. The material crystallizes with a highly porous cubic structure featuring a network of channels measuring 9 × 9 Å; it is stable up to 240 °C and has a surface area between 1300 and 1800 m^2^/g. The distance between the two metal ions (Cu-Cu) is 2.628 Å. The synthesis of Basolite C-300 is typically carried out via solvothermal or room-temperature methods. In the solvothermal approach, copper (II) nitrate or copper (II) acetate is dissolved in a solvent mixture containing ethanol (EtOH), water, and dimethylformamide (DMF), followed by the addition of 1,3,5-benzenetricarboxylic acid (btc) under controlled temperature conditions (usually around 120 °C for several hours). The resulting solid is then filtered, washed, and dried. Alternatively, room-temperature synthesis can be performed using rapid precipitation techniques, where the metal and ligand precursors are mixed under ambient conditions, yielding Basolite C-300 with slightly different textural properties [5]. Characterization of MOFs and Basolite C-300 is performed using various techniques to confirm their structure, porosity, and stability. Power X-ray diffraction (PXRD) is used to verify the crystalline nature and phase purity by comparing the obtained diffraction patterns with standard references. Fourier transform infrared spectroscopy (FTIR) helps identify the functional groups and confirm ligand coordination. Thermogravimetric analysis (TGA) assesses the thermal stability and decomposition behavior of the material. Nitrogen adsorption–desorption isotherms are used to determine the surface area and pore size distribution via the Brunauer–Emmett–Teller (BET) method. Additionally, scanning electron microscopy (SEM) and transmission electron microscopy (TEM) provide insights into the morphology and particle size distribution, while X-ray photoelectron spectroscopy (XPS) can be employed to analyze the oxidation states of the metal ions within each framework [6]. These synthesis and characterization methods ensure the reproducibility and quality of MOFs and Basolite C-300, enabling their applications in the various fields already mentioned. In this article, various techniques for the synthesis of MOFs, different methods of material characterization, MOF applications are explored.

## 2. Synthesis of MOFs and Basolite C-300

### 2.1. Conventional Methods for MOF Synthesis

Conventional methods for synthesizing MOFs include the solvothermal process, sonochemical techniques, mechanochemical approaches, microwave-assisted synthesis, continuous flow production, layer-by-layer assembly, and others. The solvothermal method, though a conventional and straightforward approach, requires extended reaction times and extreme conditions such as high temperatures and pressures. On the other hand, microwave-assisted synthesis offers a faster alternative by significantly accelerating the nucleation process. The synthesis of MOFs has garnered significant interest over the past two decades due to the possibility of obtaining a wide variety of structures with applications in the field of porous materials like zeolites and activated carbon [7,8]. One of the objectives of MOF synthesis is to establish optimal reaction conditions to achieve the formation of the compound while avoiding the decomposition of organic ligands. MOF synthesis generally involves the copolymerization of organic ligands and metal ions in a polar solvent under mild conditions (temperatures up to 200 °C and autogenous pressures not exceeding 100 atm). Other factors that dictate the properties and characteristics of the final product include the solubility of the ligands and the metal source (commonly metal salts, but metal oxides are also used), the polarity of the solvent, the ionic strength of the medium, and temperature and pressure, which play critical roles in determining the characteristics of the products [8,9]. There are a variety of procedures that have been previously studied and analyzed for obtaining these materials, such as the slow evaporation method, the diffusion method, thermal methods, microwave- and ultrasound-assisted methods, and electrochemical and mechanochemical methods. These procedures have in common a final stage consisting of washing the material with a solvent and a drying treatment with heat or under high vacuum to obtain the solid product [10].

Slow evaporation is a traditional and commonly used synthesis method for obtaining crystalline MOFs. It involves the removal of the solvent via evaporation or cooling of a saturated solution for crystal growth. This technique is subject to the following conditions: (1) crystals develop in saturated solutions, and (2) solubility increases with temperature, allowing crystals to appear during the cooling stage [10]. The technique consists of dissolving the metal source and the ligand in one or more solvents under constant stirring, and in some cases at temperatures higher than room temperature to improve solubility. The resulting solution is filtered and poured into a crystallization container, partially covered to reduce the solvent evaporation rate, aiming to control supersaturation in the system. This process facilitates nucleation and subsequent crystal growth. The products crystallize and can be obtained within minutes; however, it is more common for the process to take days to weeks or even months [8].

Diffusion is another principal method, aiming to bring different chemical species into contact so that they can slowly combine together. There are two main approaches:

1. Liquid solvent diffusion. In this procedure, two liquid layers with different densities are formed: one containing the product and the other a precipitating solvent. These layers are separated by a third solvent layer. The precipitating solvent slowly diffuses into the separated layer, and crystal growth occurs at the interface.
2. Slow diffusion of reactants. This approach involves separating the reactants with physical barriers, such as two tubes of different sizes. In some cases, gels are also used as diffusion and crystallization media, particularly to slow down diffusion and prevent material precipitation.

This method is useful for obtaining crystals suitable for single-crystal X-ray diffraction analysis, but not for non-crystalline or polycrystalline products, especially if they have low solubility [11].

Thermal methods take advantage of the self-assembly of products derived from soluble or slightly soluble precursors (metal salts and ligands). Originally used for the synthesis of zeolites, they have been adapted for the production of MOFs. These methods can be either hydrothermal or solvothermal and are considered the most effective for obtaining MOFs. These methods involve a series of processes in which chemical reactions occur under pressure and temperature conditions higher than ambient (25 °C and 1 atm). If the synthesis temperature does not exceed 100 °C, the method is termed hydrothermal, whereas at higher temperatures, the conditions are considered solvothermal [12]. The technique consists of dissolving the organic ligand and the metal salt in water, an organic solvent, or a mixture of both. The synthesis temperature eliminates the solubility constraints of the reactants within a closed space, such as a laboratory oven under autogenous pressure. The mixture of precursors and solvents is placed in vials or tubes; Teflon reactors with a steel casing are also used. The containers are sealed and heated in ovens or autoclaves for hours or even days to obtain crystals, which can be separated from the mixture by filtration, washed with an appropriate solvent, and dried at room temperature [13]. Several reaction parameters influence this type of synthesis, including the order of reagent addition, time and temperature, system agitation, reactant concentration, stoichiometry, solvent type and amount, and pH [14,15].

Microwave- and ultrasound-assisted methods are the least commonly used methods for obtaining crystalline MOFs, but they represent a highly valuable technique for carrying out synthesis extraordinarily quickly. They are effective methods for controlling the size and shape of the resulting particles. MOF synthesis can be completed in as little as 30 *s* to 2 min, with high yields ranging from approximately 30% to 85%. Additionally, particle size can be controlled by varying the precursor concentration [11].

Microwave-assisted synthesis relies on the interaction of electromagnetic waves with mobile electric charges. These charges can be polar solvent molecules, ions or electrons in solution, or ions in a solid. In solids, an electric current is generated, producing heat due to the electrical resistance of the material. In solution, polar molecules attempt to align themselves with the electromagnetic field, constantly changing orientation. Due to the direct interaction of radiation with solutions and reactants, this method is energy-efficient for heating the system and is conducted at temperatures above 100 °C, with reaction times of less than 1 h, as mentioned earlier. There are few reports detailing process variables for this method (solvent, irradiation time, reaction temperature, power level, molar ratio, reactant concentration, etc.). Ultrasound-assisted synthesis involves vibrations with a cyclic frequency between 20 kHz and 10 MHz. Since the wavelengths are larger than molecular dimensions, there is no direct interaction between molecules and ultrasonic waves, meaning no chemical reaction occurs directly. However, the accumulated energy interacts with liquids, forming alternating high- and low-pressure zones which generate bubbles up to tens of micrometers in size. Cavitation is the process in which bubbles form, grow, and collapse, creating short-lived special temperature and pressure conditions. This phenomenon induces a chemical reaction between the reactants, leading to the formation of MOFs [16,17].

Electrochemical and mechanochemical methods have been reported since 2005. The electrochemical synthesis of MOFs was reported for the first time by scientists from the chemical industry company BASF. The objective of this method is to eliminate anions such as nitrate, perchlorate, and chloride during synthesis, which is a major concern in large-scale production processes. Instead of using these anions, only metal salts are utilized, with metal ions continuously introduced into an anionic solution towards the reaction medium, which contains dissolved ligand molecules and a conducting salt. Metal deposition on the cathode is prevented by using protic solvents; however, H_2_ is produced during the process. One of the advantages of the electrochemical route is its suitability for industrial applications, as it enables a continuous process and allows for a higher solid content compared to batch reactions. Mechanical force can induce many physical phenomena as well as chemical reactions. In mechanochemical synthesis, the mechanical breaking of intramolecular bonds is the first step, followed by a chemical transformation. This method was first used in 2006 for the synthesis of MOFs. MOF synthesis reactions can be carried out at room temperature in a solvent-free system, which is an advantage from both economic and environmental perspectives. The reaction time is short, ranging from 10 to 60 min, and the products are obtained in the form of small particles [18,19].

Sonochemical synthesis is the method discussed in this section and it employs ultrasound and acoustic cavitation to facilitate the production of Metal-organic frameworks. This approach enhances chemical reactions by significantly increasing efficiency, resulting in faster reactions and thus shorter reaction times compared to traditional synthesis methods. Notably, sonochemical synthesis allows for the formation of smaller particle sizes under milder conditions, reducing the need for high temperatures and prolonged reaction durations. Additionally, this method promotes enhanced selectivity in reactions, which can lead to the formation of specific products, a phenomenon known as “sonochemical switching”. Overall, the sonochemical synthesis of MOFs represents a significant advancement in material fabrication, offering a more effective and energy-efficient alternative to conventional techniques [20]. Table 1 shows some of the most commonly used synthesis methods for the production of MOFs, as well as some reaction conditions, yield, and particle size, among other comments.

**Table 1:** A comparative table summarizing the reaction conditions, yields, particle sizes, crystallinities, and practical challenges of various MOF synthesis methods.

Synthesis Method	Reaction Conditions	Yield	Particle Size	Crystallinity	Practical Challenges	References
Hydrothermal/solvothermal technique	80–260 °C, long times (12–72 h), closed atmosphere (autoclave or Teflon-lined reactor); polar solvents such as DMF, DEF, MeOH, EtOH.	High (up to >90%), depending on precursors and time.	100 nm at >10 μm, controlled by kinetics and nucleation.	High, favored by slow diffusion and thermal balance.	Widely used technique; limitations in industrial scalability; toxic solvents; highly adjustable parameters (concentration, temperature, time, pH).	[14,15]
Microwave-assisted technique	80–200 °C, short reaction times (5–30 min); use of polar solvents with high dielectric absorption; continuous or pulsed irradiation.	High (>85%).	Uniform and lower compared to solvothermal techniques (50–500 nm).	Good to excellent, depending on the time control.	Rapid nucleation and crystallization; possible preferential orientation; requires specialized equipment; allows for significant energy savings.	[16,17]
Mechanochemical technique	Solid-phase reactions; mechanical activation by manual grinding (mortar) or automated grinding (vibrating mills or others); without the use of solvent or catalytic quantities (liquid-assisted grinding, LAG).	Variable (50–95%).	Nanometric (10–200 nm); lower dispersion.	Low to medium; can be improved by heat treatments.	Sustainable, solvent-free route; ideal for insoluble precursors; requires post-synthesis evaluation to ensure desired structure; may induce partial amorphism.	[18]
Electrochemical technique	Electrochemical cell reaction (2 or 3 electrodes); cationic metal dissolution in anode and formation of MOF in cathode, room temperature or mild heating (<60 °C); polar solvents.	Variable (40–90%).	Controllable by voltage/current and reaction time.	Good, especially in self-assembled structures on electrodes.	Allows for direct synthesis on conductive surfaces; useful for applications in sensors or catalysis; requires fine control of electrical parameters; limited to electroactive materials.	[19]
Sonochemical synthesis method	Ultrasonic irradiation in liquid medium; moderate ambient temperature (25–60 °C); intensities between 20 and 40 kHz; promotion of cavitation and nucleation.	Medium–high (60–90%).	Submicrometric, homogeneous (50–300 nm).	Good, especially in fast nucleation systems.	No severe thermal requirements; good morphological control; useful for quick synthesis.	[20]

### 2.2. New and Non-Conventional Methods for MOF Synthesis

Modified Stöber for amorphous MOFs is a method that adapts the classical Stöber technique, commonly used for the synthesis of amorphous colloids, to the field of copper-based MOFs. The process involves a vapor-based diffusion technique that controls the growth kinetics, enabling the formation of uniform MOF and coordination polymer (CP) spheres. One of the advantages of this approach is the ability to deposit the materials onto various substrates, regardless of their chemistry or morphology. It also generates amorphous MOFs, which are useful in applications where porosity and ordered structure are not essential [9].

Spray-drying methods have been reported since 2013. Maspoch et al. used for the first time a spray-drying method to synthesize MOFs in the form of spherical hollow superstructures. This method combines the atomization of solutions with continuous flow to synthesize and shape MOFs into microspheres. It is essential for industrial applications: Precursor solutions (metal salts and organic ligands) are atomized into microscopic droplets that act as microreactors. The heat evaporates the solvent, producing crystalline particles with controlled morphology [21].

Hydrothermal, microwave, and ultrasound synthesis methods are used to obtain complex architectures of MOFs and supraMOFs through the combination of copper complexes with carboxylate or polyoxoanion ligands. Hydrothermal synthesis is typically performed in autoclaves at elevated temperatures, while microwave- and ultrasound-assisted synthesis methods offer significantly reduced reaction times and improved crystal growth homogeneity [22].

The green synthesis of MOFs at room temperature is an eco-friendly approach that avoids the use of high temperatures, toxic solvents, and hazardous reagents. This method aligns with sustainable chemistry principles by reducing energy consumption and environmental impact. The advantages of this technique include lower energy consumption, reduced use of hazardous reagents, enhanced biocompatibility for biomedical applications, and environmental sustainability [23]. The green synthesis of MOFs at room temperature is a sustainable approach that minimizes energy consumption and the use of toxic solvents through methods such as mechanosynthesis, the use of eco-friendly solvents, sonication, electrochemical synthesis, and biomolecule-assisted processes. Mechanosynthesis enables the solvent-free production of MOFs, as exemplified by Basolite C-300, while the replacement of organic solvents with water has been explored in the synthesis of IRMOF-1, IRMOF-8, and IRMOF-10. Additionally, the formation of aluminum-based MOFs has been studied across a wide temperature range, including room temperature. These advancements have been reported in various sources, including studies on alternative MOF synthesis methods [24,25].

**Figure 1:**
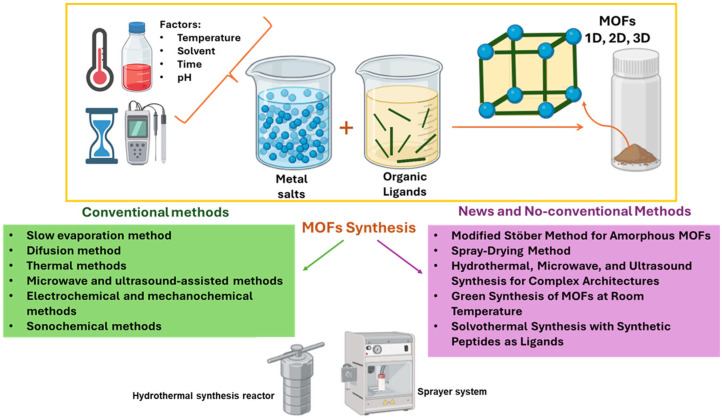
Schematic representation of general, conventional, and new and non-conventional methods (yellow, green, and pink rectangle, respectively) of MOF synthesis (created with BioRender.com).

Solvothermal synthesis is a method which involves using synthetic peptides as ligands. Copper MOFs are prepared using ligands based on synthetic peptides such as CEGH, CGH, and reduced glutathione (GHS). Synthesis is carried out at 90 °C in a mixture of water and DMF, producing particles with sizes below 600 nm. The use of peptide ligands allows for the modulation of the structural and morphological properties of the MOF [26]. Figure 1 summarizes some general, conventional, new, and non-conventional methods of MOF synthesis.

### 2.3. Basolite C-300 Synthesis and Structural Stability

**Figure 2:**
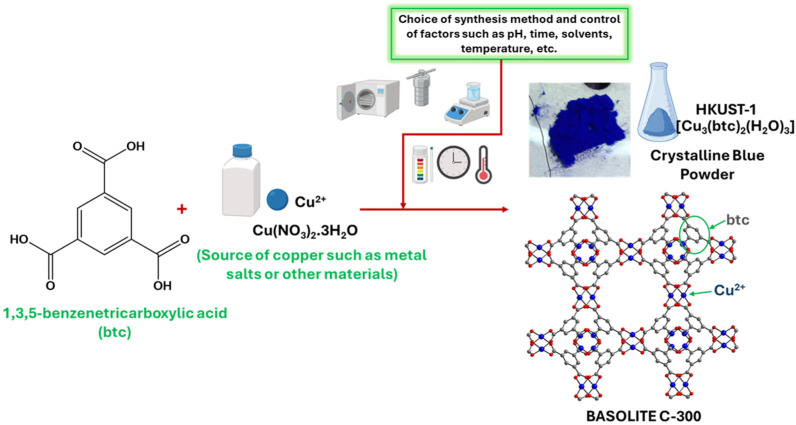
General reaction for obtaining Basolite C-300 (created with BioRender.com).

Various synthesis methods have been employed to create Cu-MOFs, utilizing a wide range of organic ligands, including ditopic, tritopic, tetratopic, hexatopic, octatopic, desymmetrized, N-heterocyclic linkers, and metalloligands. This section of the current article covers only some of the methods used to synthesize Basolite C-300, including the conventional, non-conventional, and new techniques discussed above. Figure 2 shows the reaction necessary for Basolite C-300 synthesis.

The one-step synthesis of Basolite-C300 refers to a method in which all reactants (copper source, 1,3,5-benzenetricarboxylic acid (btc), and solvents) are combined in a single stage without requiring intermediate steps such as precursors, additional pH adjustments, or phase purifications. This approach simplifies material production and improves process scalability.

Electrochemical synthesis is a method that utilizes the anodic dissolution of an copper electrode immersed in a solution containing the organic ligand btc. When an electric current is applied, the copper oxidizes and coordinates with btc to form Basolite C-300. This technique is fast, efficient, and eliminates the need for additional metal salts. However, it may require electrolytes to enhance solution conductivity and faces challenges such as electrode passivation [27].

Reflux synthesis with stirring is a method that involves mixing copper nitrate and trimesic acid in a suitable solvent under reflux conditions with continuous stirring. This technique enables the rapid and large-scale preparation of Basolite C-300, yielding high-purity octahedral crystals of uniform size [28].

Synthesis via the solvent-free method of Basolite C-300 is accomplished through a solvent-free method that enhances efficiency and minimizes the environmental impact compared to traditional techniques. This process involves using copper (II) nitrate trihydrate as the metal precursor and 1,3,5-benzenetricarboxylic acid as the organic linker. The method begins with precise weighing and mechanical grinding of the metal precursor and linker for about 15 min to ensure a uniform mixture. This mixture is then transferred to a stainless-steel autoclave and heated at 120 °C for 3–4 h, facilitating the formation of the Cu-btc framework. After cooling, the sample is washed with methanol to eliminate any unreacted materials [29].

In graphene oxide-assisted hydrothermal synthesis, graphene oxide is used as a directing agent in a hydrothermal reaction combining copper nitrate and btc. The result is a flower-like hierarchical Basolite C-300 structure integrated with graphene fragments, enhancing the material’s electrical and surface properties [30].

Table 2 shows a summary of various synthesis techniques for Basolite C-300, the metallic substrate used, solvents, and some reaction conditions [31].

Basolite C-300 demonstrates notable stability under a range of conditions, supporting its potential in applications such as gas capture and electrochemical sensing. Its structural stability is strongly influenced by the choice of synthesis method, particularly the activation procedure employed. A commonly used activation method involves washing the synthesized material with various solvents such as dichloromethane (DCM), acetone, methanol (MetOH), and ethanol–water mixtures followed by drying at approximately 100 °C. This process must be carefully controlled to prevent collapse of the crystalline framework. The activation step aims to efficiently remove water or residual solvent molecules that may reside within the pores or coordinate with the metal centers, thus exposing adsorption sites for interactions with target molecules.

Thermal analysis reveals that the material retains its structural integrity up to 250–325 °C, depending on the activation and thermal treatment conditions. Beyond this range, progressive degradation is observed, attributed to the cleavage of Cu-carboxylate bonds and subsequent formation of copper oxides. Chemically, Basolite C-300 remains stable in the presence of water, ethylene glycol, and various organic solvents, exhibiting good resistance to hydrolysis. However, it becomes unstable under strongly acidic or basic conditions, mainly due to copper leaching or protonation of the organic ligands [32].

Mechanically, its stability is constrained by its low bulk density (0.35 g/cm^3^) and the inherently fragile nature of its porous structure, which may be further weakened by residual impurities compromising the crystalline framework.

**Figure 3:**
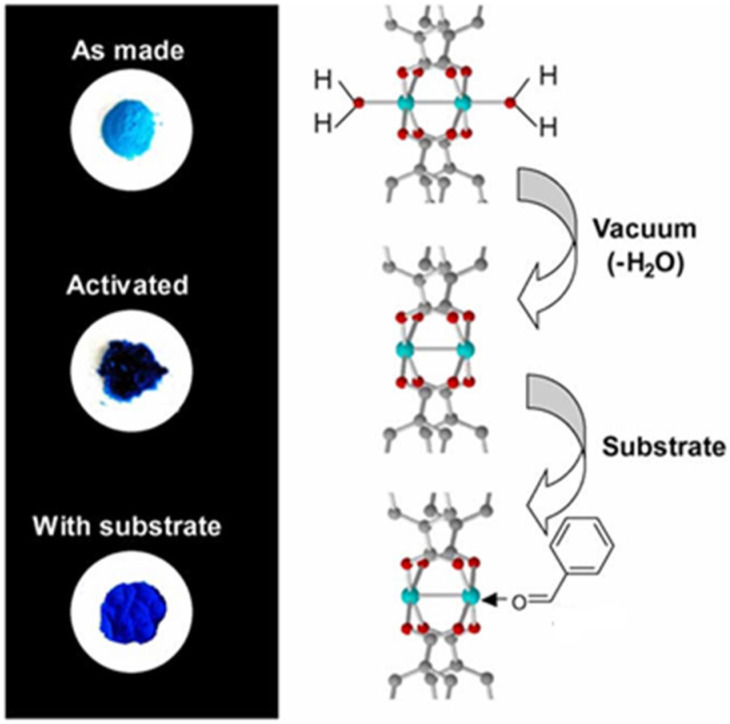
Color changes during the dehydration (vacuum activation) of Cu_3_(BTC)_2_(H_2_O)_3_·xH_2_O to form Cu_3_(BTC)_2_, and the subsequent re-adsorption of an aldehyde to form Cu_3_(BTC)_2_(C_6_H_5_CHO)_x_. [33].

From an environmental perspective, Basolite C-300 is susceptible to moisture, which induces progressive structural degradation through interactions between water molecules and open metal sites. In contrast, exposure to EtOH helps preserve the structural integrity of the framework. Water adsorption not only affects the structural integrity but also induces visible color changes (from dark violet to turquoise) which are associated with changes in the coordination state of Cu^2+^ as is shown Figure 3. The reversibility of water adsorption is limited, and the sorption capacity progressively decreases with repeated use cycles, indicating structural deterioration and a loss of porosity [33].

Overall, although Basolite C-300 exhibits generally favorable stability, optimizing its activation, storage, and operational conditions is essential to ensure maximum functional performance.

## 3. Structural Characterization and Spectra of MOFs: Basolite C-300

The characterization of Metal-organic frameworks is a complex process that requires the integration of various analytical techniques to gain a comprehensive understanding of their structure and properties. This process can be particularly challenging due to the intricate nature of MOFs, which often feature unique topologies, varying degrees of porosity, and diverse functionalities. Spectroscopic techniques, especially those capable of in situ measurements, are essential in providing detailed information about both the framework structure and host–guest interactions. These techniques are invaluable for exploring the molecular structure, bonding interactions, and the behavior of guest molecules within the pores of the MOF. For instance, vibrational spectroscopy is highly effective in revealing the nature of the bonds within the framework, identifying functional groups, and assessing the phase purity of the material. Fourier transform infrared (FTIR) spectroscopy offers insights into characteristic stretching and bending vibrations of the organic linkers and Metal-ligand interactions, while Raman spectroscopy complements this by providing information on the vibrational modes of the material. Ultraviolet–visible (UV-Vis) spectroscopy, on the other hand, provides crucial data on the electronic structure of the MOF, offering a view of its optical properties and electronic transitions. Additionally, X-ray diffraction (XRD) is essential for determining the crystalline structure and phase purity of MOFs, while electron paramagnetic resonance (EPR) allows for the analysis of paramagnetic centers and their interactions with the surrounding framework. Solid-state nuclear magnetic resonance (NMR) spectroscopy is another powerful technique that analyzes the local environment of nuclei, providing valuable insights into short-range order, local defects, and structural dynamics within the material. It is particularly useful for detecting disorders and understanding the behavior of guest molecules in the MOF. When combined, these techniques offer a comprehensive understanding of the framework, allowing for the precise characterization of the structure, functionality, and reactivity of MOFs like Basolite C-300.

### 3.1. Spectroscopic Techniques

**Figure 4:**
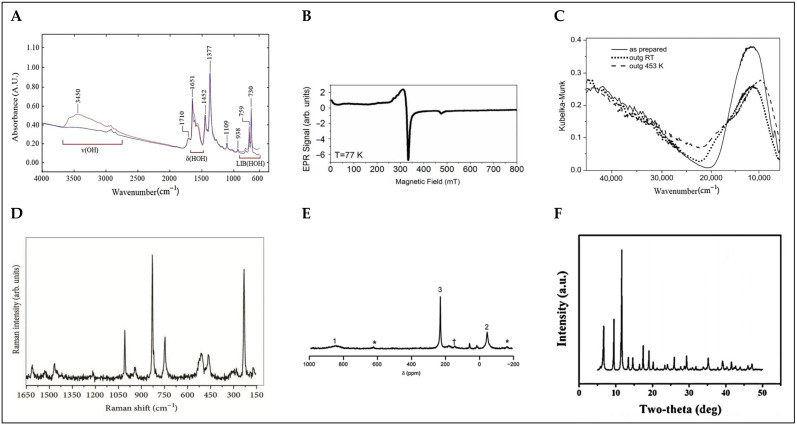
Characterization spectra of Basolite-C300: (**A**) FTIR spectra displaying characteristic vibrational modes [35]; (**B**) EPR spectra highlighting magnetic properties [43]; (**C**) UV-Vis spectra depicting electronic properties [40]; (**D**) Raman spectra showing vibrational features [39]; (**E**) NMR spectra identifying chemical environments (asterisks denote spinning sidebands and † indicates a btc-based impurity) [44]; (**F**) XRD patterns [45]. These techniques collectively offer a comprehensive analysis of the structure and properties of HKUST-1.

Vibrational spectroscopy provides important information about the structure of the molecules, their symmetry, their nature, and the strength of the bonds. It is a very powerful technique used for the characterization of organic functional groups, hydroxyl coverage, and phase purity of MOFs, as well as for studying the interaction of MOFs with guest molecules. Vibrational spectroscopy includes two main techniques, FTIR and Raman, each with their own particularities. Infrared spectroscopy methods are based on the absorption of infrared light that causes a direct transition between the vibrational energy levels of molecules, while Raman spectroscopy methods rely on the inelastic scattering of photons. The two techniques are complementary because they are sensitive to different kinds of vibrations. Readers interested in the general characterization of MOF materials and the processes of their interactions with various gases are directed to the following excellent study that provides a critically reported application of IR and Raman spectroscopies as powerful tools [34]. The infrared spectrum is conventionally divided into three subregions, each providing distinct vibrational information. The mid-infrared region (4000–400 cm^−1^) is where fundamental molecular vibrations are predominantly observed, making it the most widely used range for MOF characterization. In Basolite C-300 (see Figure 4A), this region includes the characteristic asymmetric (~1600 cm^−1^) and symmetric (~1400 cm^−1^) stretching vibrations of carboxylate (-COO^−^) groups, confirming their coordination with Cu^2+^ centers. The C-H stretching of the benzene-1,3,5-tricarboxylate (btc) linker appears around 3100 cm^−1^, while Cu-O stretching vibrations are detected below 600 cm^−1^. Additionally, changes in these bands can indicate the adsorption of guest molecules such as CO, CO_2_, NO, and H_2_, which interact with the open Cu^2+^ sites [32]. The near-infrared region (14,000–4000 cm^−1^) primarily detects overtones and combination bands, providing insights into anharmonic coupling effects and structural dynamics. In Basolite C-300, weak overtone and combination bands associated with carboxylate and Cu-O vibrations appear in this region, offering information about framework interactions and potential structural modifications upon guest adsorption [35].

The far-infrared region (400–100 cm^−1^) extends into the terahertz range and is particularly useful for studying low-energy lattice vibrations, including Metal-ligand interactions and intermolecular modes. In Basolite C-300, this region captures key vibrational features associated with the paddlewheel Cu_2_(µ-O_2_CR)_4_ structure. The Cu-O stretching modes appear around 493 cm^−1^, while vibrational bands related to Cu-Cu interactions in the dimeric framework are observed at 321, 269, and 226 cm^−1^. Upon dehydration, these bands undergo a blue shift to 504, 326, 273, and 231 cm^−1^, indicating an increase in the rigidity of the framework due to the loss of coordinated water. Additionally, the adsorption of guest molecules such as NH_3_ or CO leads to further modifications in this spectral region, reflecting changes in the local coordination environment of Cu^2+^ sites [36].

Raman spectroscopy is a powerful tool for characterizing MOFs, providing insights into structural integrity, phase purity, structural switchability, and Metal-ligand and host–guest interactions. Raman spectra are collected to investigate the influence of treatment procedures on the vibrational modes of MOFs; for example, if a sample is exposed to humid air and treated with different solvents, then the results are compared with those of the pristine material, with some characteristic bands expected to change after the treatments [37]. When DMF, dimethyl sulfoxide (DMSO), MeOH, acetone, or tetrahydrofuran (THF) bind to the HKUST-1, the Cu-Cu Raman stretch is around 180 cm^−1^. For acetonitrile (MeCN), the Raman shift is at 184 cm^−1^, whereas for EtOH and DCM, the shifts are at 189 and 214 cm^−1^, respectively. In addition, the thermodynamics of solvent binding in MOFs can be studied by monitoring the equilibria of the coordinatively unsaturated site (CUS)-bound solvent in mixed solvent systems via Raman spectroscopy [38]. In Basolite C-300 (see Figure 4D), Raman spectroscopy reveals characteristic vibrational bands associated with its paddlewheel Cu_2_(µ-O_2_CR)_4_ structure, including Cu-Cu stretching modes at 173 and 191 cm^−1^, Cu-O stretching vibrations at 502 and 449 cm^−1^, benzene ring C-H out-of-plane bending at 745 and 828 cm^−1^, symmetric C=C stretching at 1006 cm^−1^, asymmetric and symmetric O-C-O stretching at 1544 and 1460 cm^−1^, and benzene C=C stretching at 1616 cm^−1^. Additionally, Raman spectroscopy is crucial for monitoring the structural stability of Basolite C-300 under environmental conditions, particularly its sensitivity to moisture, which induces hydrolysis of Cu-O bonds. This process results in a blue shift of the Cu-Cu stretching mode, indicating distortion of the paddlewheel structure, a decrease in the intensity of the Cu-O stretching band at 502 cm^−1^, reflecting coordination changes and framework degradation, and a red shift in the O-C-O stretching bands, signifying modifications in the carboxylate coordination environment. Systematic Raman measurements indicate that for Basolite-C300, exposure to moisture times longer than 20 days results in irreversible processes of decomposition taking place [39].

UV-Vis spectroscopy is a key technique for analyzing the electronic properties and structural characteristics of MOFs. The UV-Vis spectrum of Basolite C-300 (see Figure 4C) reveals significant absorption bands that provide insights into its electronic structure. A strong ligand-to-metal charge transfer (LMCT) transition is observed around 30,000 cm^−1^, which corresponds to electron transfer from the oxygen atoms of the carboxylate ligands to the Cu^2+^ metal centers. This transition is a critical feature of the material’s electronic behavior. Additionally, the d-d transitions of the Cu^2+^ centers, which result from the Jahn–Teller distortion in the Cu paddlewheel structure, are seen in the UV-Vis spectrum at 11,900 cm^−1^. These transitions indicate the distortion from perfect octahedral geometry and modification in the symmetry around the copper centers. Upon thermal treatment, these d-d transitions undergo shifts, and the intensity of the absorption band increases, demonstrating changes in the local symmetry around the Cu^2+^ sites due to water removal and the consequent coordinative unsaturation. These features are crucial for understanding the electronic configuration and reactivity of Basolite C-300, particularly its ability to interact with guest molecules in various applications [40]. UV-Vis spectroscopy is also valuable for monitoring structural and electronic modifications in Basolite C-300 upon environmental exposure or functionalization. Studies have shown that the adsorption of guest molecules, such as dyes or gases, can induce shifts in the absorption bands, revealing host–guest interactions. Furthermore, hydration and dehydration processes can alter the intensity and position of the absorption peaks, indicating changes in the coordination environment of Cu^2+^ centers. The ability to track these variations highlights the role of UV-Vis spectroscopy in evaluating the stability and performance of Basolite C-300 in various applications, such as catalysis, sensing, and pollutant adsorption [41].

Electron paramagnetic resonance (EPR) spectroscopy is a powerful technique for analyzing the local electronic environment of paramagnetic centers in Metal-organic frameworks, particularly those containing transition metals with unpaired electrons. In materials like Basolite C-300, which features Cu^2+^ ions in a paddlewheel structure, EPR provides crucial insights into the oxidation state, coordination environment, and overall framework stability. The Cu^2+^ ions in this structure exhibit antiferromagnetic coupling due to superexchange interactions through the carboxylate bridges, resulting in a characteristic EPR signature of weakly coupled spin S = 1/2 systems. For HKUST-1 (see Figure 4B), EPR spectroscopy reveals distinct signals corresponding to Cu^2+^ centers within the paddlewheel units. The primary signal, observed in the g-range of approximately 2.07 to 2.32, is associated with Cu^2+^ centers and offers valuable information about the metal’s electronic environment. Minor resonances at ~12 mT and ~470 mT correspond to triplet states (S = 1), which arise from weak interactions between Cu^2+^ pairs in the paddlewheel structure. Upon exposure to moisture, significant changes in the EPR spectrum are observed, attributed to the hydrolysis of Cu-O bonds. This process results in the gradual disappearance of the Cu^2+^ paddlewheel signal and the emergence of new paramagnetic species, specifically E’_1_(Cu) and E’_2_(Cu), which correspond to copper sites with reduced coordination and structural modifications. The E’_1_(Cu) species appears after around 20 days of air exposure, reflecting partial hydrolysis of the carboxylate bridges, while the E’_2_(Cu) species emerges after extended exposure, indicating further degradation of the framework. The ability to monitor these structural changes in real time makes EPR particularly valuable for studying the stability of Basolite C-300 under different environmental conditions. The high sensitivity of EPR allows for the detection of atomic-level modifications, providing insights into defects, degradation mechanisms, and the effects of guest molecule interactions on the integrity of the framework. Such information is essential for applications involving gas adsorption, catalysis, and long-term material durability, where the stability of metal centers plays a critical role. Therefore, EPR spectroscopy is an indispensable tool for understanding the dynamic behavior and structural evolution of HKUST-1 under various operational conditions [42,43].

Nuclear magnetic resonance (NMR) spectroscopy is a well-established method used for the investigation of various types of porous materials. Solid-state NMR spectroscopy is an important tool for the study of the structure dynamics and flexibility of MOFs, as well as for the characterization of host–guest interactions with adsorbed species, such as xenon, carbon dioxide, water, and many others, including in situ studies of catalytic reactions and diffusion processes. Solid-state NMR is able to identify structural parameters and detect dynamical effects. It is capable of revealing the presence of mobile substructures or molecules. Solid-state NMR experiments for the detection of thermal motions/exchange processes, such as one-dimensional exchange spectroscopy by sideband alternation or dipolar center band-only detection of exchange, which were previously designed for other materials, may also become useful for MOFs. Note that the latter experiment is capable of detecting slow motions from milliseconds up to seconds. For the investigation of the framework itself, ^1^H and ^13^C NMR are often used (for Basolite C-300, see Figure 4E). In addition, other nuclei such as ^27^Al, ^71^Ga, ^45^Sc, and ^67^Zn can be studied by magic-angle spinning (MAS) NMR spectroscopy in order to detect the environment of the central metal atom. Solid-state NMR spectroscopy is, furthermore, extremely helpful in characterizing the interactions between the framework and adsorbed species (host–guest interactions including structural changes). For example, ^129^Xe NMR spectroscopy of adsorbed xenon is an excellent method for the study of porous materials because a variety of NMR parameters, in particular the ^129^Xe chemical shift, line width, chemical shift anisotropy, and longitudinal relaxation time T1, are influenced by structural parameters such as pore size, pore shape, the composition of the pore walls, and dynamics. The adsorption of other molecules such as carbon dioxide and water can also be studied by using NMR spectroscopic techniques [44].

X-ray-based spectroscopic techniques provide crucial insights into the electronic structure, oxidation states, and local coordination environments in MOFs. In Basolite C-300, these techniques are essential for understanding the valence state of Cu^2+^, Metal-ligand interactions, and framework stability under different conditions (see Figure 4F) [45]. X-ray Photoelectron Spectroscopy (XPS) enables surface-sensitive analysis of the chemical composition, while X-ray Absorption Spectroscopy, including X-ray Absorption Near-Edge Structure (XANES) and Extended X-ray Absorption Fine Structure (EXAFS), analyzes the local environment of metal centers at the atomic level [46,47,48]. XPS studies of Basolite C-300 reveal characteristic Cu 2p_3_/_2_ and Cu 2p_1_/_2_ peaks at ~934.2 eV and ~954.5 eV, respectively, along with shake-up satellite features around 940–943 eV, confirming the presence of Cu^2+^ in the framework. The O 1s spectrum exhibits distinct peaks at ~531.5 eV (C=O/O-C=O from carboxylate groups) and ~533.1 eV (Cu-O-C bonds), indicating strong coordination between btc ligands and Cu^2+^ centers. Functionalization with ethylenediamine or other amine-based molecules leads to notable shifts in the N 1s spectrum, with peaks emerging at ~399.5 eV, corresponding to N-Cu interactions, providing evidence of post-synthetic modifications affecting the coordination sphere of Cu^2+^ [47]. XANES studies at the Cu K-edge (~8979 eV) confirm the divalent oxidation state of Cu in HKUST-1, with edge positions matching those of CuO reference compounds. Upon exposure to moisture, significant spectral changes are observed, including an increase in the intensity of the 1s → 3d pre-edge transition (~8978 eV), indicating modifications in the electronic structure of Cu^2+^ centers due to water adsorption. The white line intensity at ~8998 eV also increases with prolonged air exposure, reflecting alterations in the ligand field and coordination environment around Cu^2+^ ions. These findings highlight the sensitivity of Basolite C-300 to humidity and its potential for structural transformations under ambient conditions [48]. EXAFS analysis provides further insights into the local coordination geometry of Cu^2+^ centers. In pristine HKUST-1, Cu^2+^ ions exhibit a paddlewheel dimer structure, with Cu-Cu distances of ~2.64 Å and Cu-O distances of ~1.96 Å. After 20 days of air exposure, a slight elongation of Cu-Cu distances (~2.63 Å) and an increase in Cu-O distances (~1.97 Å) indicate hydration-induced distortion of the framework. These structural changes are reversible upon reactivation under vacuum at 400 K, demonstrating the dynamic nature of Cu^2+^ coordination within HKUST-1 [49]. The combination of XPS, XANES, and EXAFS provides a comprehensive understanding of the electronic and structural properties of Basolite C-300, particularly in response to environmental factors. These techniques are essential for assessing the stability of Basolite C-300 in applications such as gas storage, catalysis, and adsorption-based separations.

### 3.2. Complementary Techniques

The characterization of MOFs such as Basolite C-300 requires a multi-technique approach to fully understand their structural, electronic, and textural properties. While spectroscopic methods provide valuable insights into the chemical bonding and electronic environment of the framework, complementary techniques such as Brunauer–Emmett–Teller (BET) analysis, thermal analysis, and electron microscopy are essential for obtaining a comprehensive understanding of the MOF’s structure and performance. These methods enable the determination of crystallinity, porosity, stability, and morphology, which are crucial for evaluating the potential applications of MOFs in catalysis, gas storage, and adsorption-based separations.

**Figure 5:**
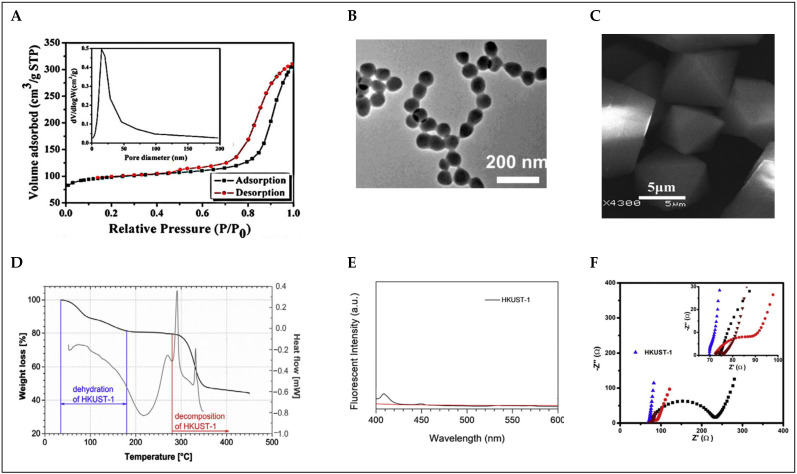
Complementary and analytical multi-technique approaches to Basolite C-300 characterization: (**A**) BET analysis [45]; (**B**) TEM image of 200 nm [51]; (**C**) SEM image of 5 µm [45]; (**D**) TGA (under nitrogen) and DSC analysis [53]; (**E**) fluorescence spectra [54]; (**F**) EIS measurements [57].

Brunauer–Emmett–Teller (BET) analysis is a widely used method for determining the specific surface area of porous materials. This technique relies on the adsorption of a gas, typically nitrogen, on the material’s surface at low temperatures (usually 77 K). The resulting adsorption isotherm is used to calculate the total surface area of the material, providing a quantitative measure of its adsorption capacity. The BET method is especially valuable for characterizing materials such as Metal-organic frameworks like Basolite C-300 (see Figure 5A) [45], zeolites, activated carbon, and other porous substances that feature large internal surfaces. In addition to specific surface area, BET analysis also enables the determination of textural properties, such as pore volume and pore size distribution, which are critical for applications in gas storage, catalysis, separation, and energy storage. For example, in previous reports, Basolite C-300 exhibited a BET surface area of 1055 m^2^/g, while another study reported a surface area of 1615 m^2^/g for Basolite C-300 under optimized conditions. These values highlight the importance of synthesis methods and activation processes in optimizing the surface characteristics of porous materials [49,50].

Scanning electron microscopy (SEM) and transmission electron microscopy (TEM) are invaluable tools for visualizing the morphology and internal structure of MOFs such as HKUST-1 (see Figure 5B,C). These electron microscopy techniques provide detailed images that reveal the crystal morphology, size distribution, porosity, and structural integrity of the framework. In the case of Basolite C-300, SEM is often used to observe the overall morphology of the crystals, which typically appear as well-defined octahedral shapes. The SEM images of Basolite C-300 crystals show uniform distribution and well-defined crystal facets, and are thus essential for understanding the material’s structural uniformity and its potential for various applications such as gas storage and catalysis [45]. TEM, on the other hand, provides high-resolution images that allow for the observation of the internal structure of Basolite C-300 at the nanoscale. TEM images of HKUST-1 reveal the porous network within the crystals, confirming the material’s mesoporous and microporous characteristics. Nanoscale imaging also aids in studying the crystallinity and phase distribution within the material, ensuring the purity and structural integrity of the synthesized framework [51]. These electron microscopy techniques, in conjunction with other characterization methods like X-ray diffraction (XRD) and nitrogen adsorption/desorption analysis, help to comprehensively assess the quality, size distribution, and porosity of HKUST-1 crystals, which are critical for their performance in adsorption, catalysis, and separation processes.

Thermogravimetric analysis (TGA) and differential scanning calorimetry (DSC) are essential techniques for evaluating the thermal stability and decomposition behavior of MOFs. TGA measures the weight loss of a sample as a function of temperature, providing insights into the material’s stability, solvent content, and thermal degradation. For HKUST-1, TGA results reveal that the framework undergoes dehydration at temperatures around 180 °C, with a total weight loss corresponding to the removal of water molecules from the copper centers. This loss is indicative of the thermal stability of the material, which is reported to be stable up to 280 °C before disintegration begins. In addition to TGA, DSC is used to study the heat flow into or out of a sample as it is heated or cooled. For HKUST-1, DSC analysis shows an endothermic peak between 100 and 280 °C, corresponding to the dehydration process, while exothermic peaks after 280 °C indicate the decomposition of the framework into Cu_2_O and CuO. These combined thermal analyses provide a comprehensive understanding of the thermal stability and decomposition mechanisms of HKUST-1, which is critical for evaluating its performance in applications such as gas adsorption and separation [52,53]. Figure 5D shows the corresponding TGA and DSC curves.

### 3.3. Analytical Multi-Technique Approaches

In addition to conventional characterization methods, several advanced techniques are crucial for analyzing specific properties of MOFs. These specialized techniques offer deeper insight into characteristics, such as optical behavior, electronic structure, and material stability under varying environmental conditions. Similarly, techniques such as UV-Visible spectroscopy, Raman spectroscopy, and electron microscopy can be used to explore various aspects of the material’s behavior, including light absorption, structural integrity, and surface characteristics. This section explores these and other complementary methods, discussing their ability to measure and characterize unique properties of HKUST-1, which are essential for optimizing its performance in applications like catalysis, gas storage, and sensing.

Spectrofluorometry is a technique used to study the luminescent properties of materials. This method involves measuring the fluorescence emission of a material after it has absorbed light at a specific wavelength. In the case of HKUST-1, spectrofluorometry can provide valuable insights into its optical properties, particularly in relation to its metal centers (Cu^2+^) and organic ligands (see Figure 5E). For Basolite C-300 and its composites, fluorescence quenching or enhancement can occur due to interactions between the metal centers and guest molecules. For example, when HKUST-1 is incorporated with carbon dots (CDs), the fluorescence properties are altered due to photo-induced electron transfer (PET) between the CDs and Cu^2+^ centers. In the absence of water, the fluorescence is weak due to PET, but upon exposure to water, the fluorescence intensity can be “turned on,” indicating the sensitivity of HKUST-1 to water molecules. The use of spectrofluorometry in the detection of water in organic solvents, such as ethanol and acetone, highlights its practical application in environmental monitoring, where changes in fluorescence intensity can be used to quantify trace amounts of water. This technique offers a simple, fast, and sensitive method for real-time detection, especially when combined with smartphone-based detection systems for portable use [54].

The magnetic susceptibility of MOFs can provide valuable insights into their electronic and magnetic properties. In the case of Basolite C-300, which features copper-based paddlewheel motifs (Cu_3_), the magnetic behavior arises from the interactions between Cu^2+^ ions in the framework. HKUST-1 exhibits an antiferromagnetic (AFM) ground state, where the spin configurations of adjacent copper ions are oppositely aligned, leading to the cancellation of the overall magnetic moment. This AFM ordering is primarily due to the strong antiferromagnetic interaction within the Cu/Cu paddlewheels, meaning that no significant magnetic interactions occur between the paddlewheels. Magnetic susceptibility measurements can be performed using a vibrating sample magnetometer (VSM) or a SQUID magnetometer, which detects the response of the material to an external magnetic field. These techniques provide data that allow for the determination of the material’s susceptibility, and in the case of HKUST-1, studies have shown that its magnetic properties change with temperature, especially above 100 K, where an increase in susceptibility can be observed. At higher temperatures, HKUST-1 may transition between different spin states, such as from the antiferromagnetic state to the ferromagnetic (FM) state, depending on external conditions like temperature or pressure. This behavior is attributed to the spin flipping and changes in the Cu/Cu distance, which can be induced by external stimuli, such as the presence of gases or mechanical pressure. Magnetic susceptibility analysis of Basolite C-300 is useful for understanding its potential applications in areas like gas sensing, catalysis, and electronics, as the magnetic properties of the material can be tuned to respond to external fields or changes in the environment [55].

Electrochemical impedance spectroscopy (EIS) is an essential technique for evaluating the electrochemical properties of materials like Basolite C-300, particularly when considering its use in sensors, catalysis, and energy storage devices. EIS provides a comprehensive analysis of the resistance, capacitance, and charge transfer dynamics of a system, helping to characterize its ability to conduct and transfer electrons efficiently. For HKUST-1, EIS is used to understand the electronic conductivity and electrocatalytic activity of the framework (see Figure 5F), which is critical for applications like sensors or batteries where electron transfer plays a vital role. Typically, the impedance spectra from EIS measurements reveal key information about the charge transfer resistance (Rct) and the double-layer capacitance (Cdl), which are influenced by the structural and electronic properties of the MOF. In an example application, HKUST-1/GONRs/GCE (a composite electrode of HKUST-1 with graphene oxide nanoribbons) was studied using EIS to enhance the electrochemical performance of the sensor for detecting imatinib, an anti-cancer drug. The results showed a significant improvement in charge transfer efficiency due to the incorporation of conductive graphene oxide, which improved the electron flow in the system. EIS measurements at different stages of electrode modification demonstrated how the presence of graphene oxide nanoribbons reduced the charge transfer resistance, making the electrode more efficient for electrochemical sensing. The Nyquist plots, which are a typical output of EIS, are analyzed to evaluate the impedance behavior of the HKUST-1 framework, revealing how well it interacts with the electrolyte and how it can facilitate electron transfer. This analysis can also highlight the impact of various modifications to the HKUST-1 structure, such as the introduction of conductive components like graphene oxide. Then, EIS provides valuable insights into the electrochemical properties of HKUST-1, helping to optimize its performance in various applications by improving charge transfer capabilities and electrochemical response [56].

## 4. Applications of MOFs and Basolite C-300

**Figure 6:**
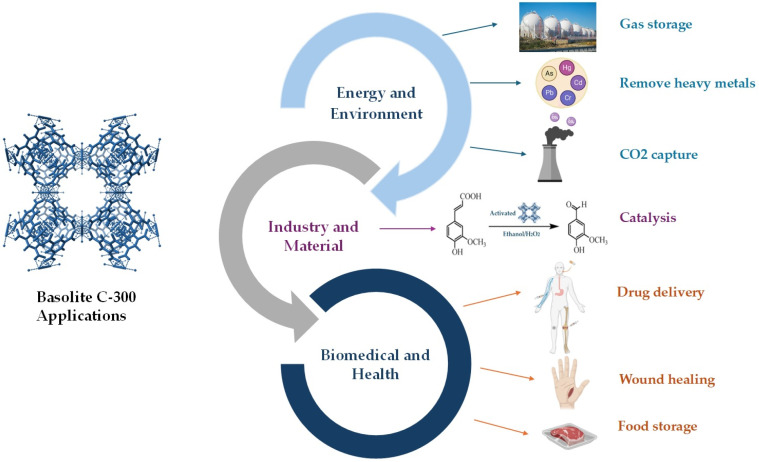
Applications of Basolite C-300 in various fields. Created with BioRender.com.

In recent decades, significant advancements have been achieved in developing novel MOFs and understanding their unique properties. This trend has exhibited an even greater pace in the past few years. Since their discovery, these materials have been used in various technological fields, including environmental pollution remediation, gas storage separation, catalysis, electrochemical sensing, energy storage and batteries, medical diagnosis, drug delivery, and others [58,59,60,61,62]. Among the many MOFs, Basolite C-300 has attracted significant interest due to its properties and versatility across various scientific and industrial applications. Its stability, combined with its ability to enhance the performance of various formulations, improves the dispersion of compounds and maintains stability under demanding conditions, making it an ideal candidate for numerous uses [63,64,65]. Figure 6 shows the application of Basolite C-300 in various fields.

### 4.1. Industry and Materials

MOFs represent an emerging class of microporous materials characterized by a high surface area, structural flexibility, and topological diversity. These structures have emerged as promising candidates for heterogeneous catalysis in industrial applications due to their stability as hybrid materials which are synthesized, optimized, and functionalized through the incorporation of organic ligands and metal ions with well-defined coordination geometries. In particular, MOFs have demonstrated significant potential in heterogeneous catalysis due to their hybrid nature, structural stability, and the ability to be synthetized and functionalized in a controlled manner. Furthermore, they are gaining recognition as a new class of active materials for organic reactions. Integrating MOFs with metal nanoparticles now allows for the fabrication of advanced hybrid catalytic materials that demonstrate enhanced recyclability, reparability, and activity for specific organic processes [66,67,68]. MOFs have proven promising materials for various electrochemical applications in recent years due to their porous structure and high specific surface area, which enable efficient charge storage and transfer. In particular, the incorporation of active metals and/or ligands to introduce redox and catalytically active sites imparts the necessary functions to MOFs for electrochemical sensing. These properties make them ideal for batteries, supercapacitors, and electrochemical sensors. Furthermore, there have been continuous efforts to design, synthesize, and make engineering use of green MOFs [69,70,71].

Basolite C-300 is one of the compounds widely used in catalysis. Its high surface area and metal centers are crucial in catalytic processes. The catalytic activity of this material is primarily attributed to the copper ions (Cu^2+^/Cu^+^) integrated into its framework which serve as active sites for various catalytic reactions. This material has demonstrated exceptional performance in oxidation and reduction processes, particularly in CO_2_ reduction, where it facilitates the conversion of carbon dioxide into valuable chemical products, showing potential for applications in clean energy technologies. Additionally, its ability to catalyze the degradation of organic pollutants positions it as an ideal candidate for environmental protection, particularly in water treatment and air purification. The high surface area and tunable porosity of Basolite C-300 further enhance its efficacy in organic synthesis, enabling selective reactions such as hydrogenation and dehydrogenation in liquid-phase catalysis. These combined properties make Basolite C-300 a versatile material with significant potential for sustainable processes in both industrial and environmental applications [41,72,73,74,75,76].

### 4.2. Energy and Environment

Gas storage and separation are key processes in industrial, environmental, and energy applications, ensuring pure raw materials and limiting pollutant emissions. In this way, gas separation and storage technologies are key to environmental and economic sustainability. MOFs have shown significant advancements in gas storage and separation in recent years. Compared with conventional porous materials such as activated carbons, silicas, and zeolites, MOFs possess unique structural attributes, including porosity, extensive surface area, and adjustable functionality, making them highly promising for gas storage and separation processes [77,78,79]. Specifically, Basolite C-300 has been investigated for its ability to remove heavy metals and other organic pollutants from aqueous environments and air. Due to its structure, it can efficiently capture toxic metal ions such as lead, mercury, and cadmium, making it a valuable material for water purification. Furthermore, its capacity to adsorb gases such as carbon dioxide (CO_2_), hydrogen (H_2_), and various other gases including hydrocarbons and noble gases positions Basolite C-300 as a promising material for addressing challenges in clean energy and environmental sustainability [53,79,80]. Notably, it has been shown to adsorb large quantities of methane at low pressures, positioning it as a promising material for natural gas storage. On the other hand, it has been found that copper-based MOFs can incorporate all factors influencing gas adsorption and separation through the selection of organic ligands. Additionally, CU-MOFs more easily create open metal sites due to the weak Cu-O bond in the CU–water complex, generating strong binding energy that enhances gas storage and separation. Other applications of MOFs include the detection of small molecules, pesticides, and organic compounds gases, as well as pollution monitoring through key biomolecules [81,82,83,84,85].

The large surface area, distinctive ordered structure, and exceptional electrical conductivity of MOF materials also position them as promising candidates for energy storage. Due to its high specific surface area, large pore volume, and excellent thermal stability, Basolite C-300 is also being investigated for use in supercapacitors and lithium-ion batteries, which are essential for both short- and long-term energy. Its ion exchange capacity, rapid discharge capability, and electrical conductivity, coupled with its porous structure, make it a promising material for energy storage; such properties are key factors in the development of portable electronics, electronic vehicles, and renewable energy networks [86,87,88].

In addition, MOFs have emerged as highly effective materials for environmental remediation due to their high surface area, tunable porosity, and chemical versatility. These properties enable MOFs to efficiently adsorb various contaminants, including heavy metals, organic pollutants, and colorants. Heavy metals, such as lead, mercury, and cadmium, are highly toxic and can accumulate in the environment, posing significant risks to both ecosystems and human health. MOFs have demonstrated remarkable capacity in adsorbing these metals from contaminated water, offering an efficient method for their removal and helping to mitigate their environmental impact. Organic pollutants, such as pesticides, pharmaceuticals, and industrial chemicals, are commonly present in wastewater and can have severe detrimental effects on aquatic life and human health [84,89,90,91,92,93,94]. Due to their high surface area and tunable pore structures, MOFs are capable of adsorbing and capturing a wide range of organic pollutants, making them highly effective tools for wastewater treatment. Colorants, particularly those used in the textile and dye industries, are difficult to remove and can persist in water, contributing to environmental pollution and posing health risks. MOFs have shown significant promise not only in adsorbing these colorants but also in degrading them, aiding in the decolorization of wastewater and enhancing water quality. On the other hand, HKUST-1 has proven to be effective as an adsorbent for various types of textile dyes, including direct, acid, basic, and vinyl sulfonic reactive dyes. Moreover, methylene blue pollutants are toxic, carcinogenic, and mutagenic, degrading water quality and posing significant risks to human health. HKUST-1 and its composites, particularly those with hierarchical pore structures, have been demonstrated to be efficient adsorbents for the removal of methylene blue [95,96,97].

### 4.3. Biomedical and Health

Due to their unique combination of redox, photochemical, and electrical properties and the catalytic activities of Cu^2+^, copper-based MOFs (Cu-MOFs) have been thoroughly examined in various biomedical fields. Thanks to the exceptional features of MOFs, including their high porosity, extensive surface area, large pore size, nanometer-scale size, biocompatibility, and biodegradability, these materials have great potential in biomedical applications, including drug delivery, biosensing, bioimaging, wound healing, biocatalysis, and others [98,99,100].

Currently, the development of efficient systems for drug delivery is a central area of research, aiming to ensure favorable outcomes through the use of safe and reliable materials. Conventional carriers such as nanoemulsions, carbon dots, and liposomes have been extensively studied; however, many of these systems face challenges, including low drug loading capacity, potential toxicity, and poor control over drug release kinetics. High drug loading capacity, low toxicity, and good biocompatibility are fundamental aspects of drug delivery applications, and their limitations can be overcome through advancements in the development of MOFs. Indeed, magnetic drug delivery enables targeted drug transport to a specific organ or area using magnetically sensitive materials coated with a drug-loaded matrix. Magnetic MOF nanoparticles deliver the drug to the treatment site without affecting other cells. In this context, the synthesis of MOFs such as Cu-BCT (HKUST-1), ZIF-8, and MOF-5 has been explored for drug delivery. Additionally, a self-sustaining nanosystem was created by encapsulating chlorin e6-modified ADNzima (C6-ADNzima) in ZIF-8, targeting gene therapy and photodynamic therapy. ADNzima was efficiently delivered through ZIF-8 nanoparticles without enzymatic degradation in cancer cells [101,102,103,104].

The highly ordered crystalline structures of MOFs can be considered organic building blocks that could be assembled in various ways to provide enormous flexibility in pore size, structure, and other properties. Due to this flexibility, MOFs have shown great potential in food safety detection and analysis, the removal of pesticide residues and veterinary drugs, and food monitoring and preservation. Additionally, these properties have served as a basis for exploring the potential of MOFs in food packaging. One application of MOFs in this field is the adsorption of ethylene, a plant hormone, to control ripening and extend the shelf life of fresh products. Some studies have reported the potential of MOFs as ethylene adsorbents to extend the shelf life of bananas and, likely, other fresh products [105,106,107,108,109,110,111,112,113].

Recently, Basolite C-300 has been employed as an active agent to prolong the shelf life of food and ensure safety. Organic frameworks in active packaging act as oxygen scavengers, antimicrobials, moisture absorbers, and ethylene scavengers. The removal of free fatty acids and peroxy compounds can be performed via cobalt, iron, and zinc-based Metal-organic frameworks. Cu-MOF has scope in the development of hydrogen peroxide detection sensors. In the food industry, hydrogen peroxide is widely used for the sterilization of equipment and preservation. Basolite C-300 can also serve as host molecules for the controlled release of active organic molecules such as 1-methyl cyclopropene (1-MCP), hexanal, and EtOH, among others. Such host–guest systems can be deployed for active packaging applications. However, the toxicity and stability of MOF materials are critical factors for their applications, especially in food-related uses. Parameters affecting the toxicity of MOFs include metal ions, organic linkers, the solvent used for the synthesis, their size composition, and their solubility, degradation, and stability in biological systems. Zinc and iron metals are considered the least toxic metals, while cadmium is the most toxic metal for biological and food-related applications of MOFs. Other applications of HKUST-1 include the in situ modification of natural fabrics for controlled release of insect repellents, such as N, N-diethyl-3-mehtylbenzamide. This application enhances the functionality of textiles, providing an effective solution for prolonged insect protection [113,114,115,116,117,118,119,120].

## 5. Summary Discussion and Final Conclusions

The current limitations of Basolite C-300 and its MOF analogues are associated with several aspects, including poor hydrolytic stability, structural collapse upon guest desorption, relatively low electrical conductivity, etc. Future research must continue addressing these issues. In addition, the goals of better understanding framework organization using instrumental chemical elucidation, increasing yield, reducing costs, and facilitating material reuse to minimizing environmental impact represent an area of opportunity. The chemical structure of MOFs is usually dictated by the geometry and connectivity of organic linkers. Modifying the geometry, length, ratio, and functional groups of linkers can fine-tune the size, shape, and surface properties of an MOF to suit specific applications. These challenges have prompted the exploration of new methods and conditions of synthesis, aimed at improving the purity and crystallinity of pristine materials. In contrast to traditional matrixes, MOFs possess thousands of different structures which can be prepared by changing a wealth of customizable organic ligands and metal nodes. Then, the ongoing and intriguing challenge lies in discovering serendipitous combinations of metal ions and ligands that promote the right connectivity through the chemical bonding states as covalent interactions, coordination bonds, Van der Waals forces, etc. Furthermore, to address these challenges, new MOF-based materials have emerged, such as MOF composites, Metal-organic polyhedra (MOPs), molecularly imprinted polymers (MIPs), and others.

Due to their highly porous structure, flexibility, and ability to be designed with an almost infinite variety of compositions, MOFs have been the subject of extensive research in recent years. Their versatility enables them to play a key role in various applications, ranging from gas storage and capture to environmental remediation and biomedicine. This review presents an overview of MOFs and Basolite C-300, discussing different synthesis methods, structural and spectral characterization, and applications. Among the most studied MOFs, Basolite C-300 stands out due to its unique three-dimensional structure and exceptional adsorption properties, making it an ideal candidate for a wide range of applications. Its high porosity, structural stability, and ability to interact with diverse molecules further enhance its potential for applications in industries of material science, electrochemistry, gas storage, environmental remediation, energy, and biomedicine. The synthesis of Basolite C-300 has been the focus of various studies, and although significant progress has been made in optimizing conditions, its preparation remains a challenge. Improvements in synthesis methods have led to better control over the material’s structure and functionality, facilitating its integration into areas with significant industrial and scientific impact. Regarding its structure, Basolite C-300 is defined as a three-dimensional network organized through Metal-organic linkages, offering an environment conducive to the adsorption of diverse molecules. Spectroscopic studies, such as X-ray diffraction, infrared spectroscopy, and nuclear magnetic resonance, have provided a deeper insight into the material’s architecture, allowing for a more precise evaluation of its performance in specific applications. In terms of applications, Basolite C-300 has shown great potential in several fields. In industrial and materials applications, it has served as an adsorbent in gas purification and separation processes. In electrochemistry, it has demonstrated excellent energy storage capability, acting as an anode material in batteries. On the other hand, its gas adsorption and desorption capabilities show suitability for CO_2_ storage and capture in the energy industry and the environment. Moreover, its ability to capture pollutants has made it a valuable tool for addressing water and air contamination. Furthermore, in the biomedical field, its properties as support for controlled drug release, food storage, and medical diagnostics are being explored. Basolite C-300 has emerged as a multifunctional material with significant potential across various disciplines. Although challenges remain, particularly regarding its long-term stability and toxicity, its ongoing study opens new possibilities for more sustainable and innovative technologies. Future research on Basolite C-300 will likely lead to new applications and advancements in the previously mentioned fields, solidifying its role as a key material.

## Figures and Tables

**Table 2:** The synthesis of C-300 Basolite using various methods. In all cases, the organic ligand used is btc (1,3,5-benzenetricarboxylic acid) [31].

Synthesis Method	Metallic Source	Solvent Used	Reaction Conditions	Comments
Hydrothermal/solvothermal technique	Cu(NO_3_)_2_·3H_2_O	Ethanol and water	25–80 °C 12 h	Nanoporous material
Hydrothermal/solvothermal technique	Cu(CH_3_COO)_2_·H_2_O	DMF	120 °C 20 h	Octahedral-shaped morphology
Hydrothermal/solvothermal technique	Cu(NO_3_)_2_·3H_2_O	Ethanol and DMF	130 °C 24 h	Antibacterial agent
Electrochemical technique	Cu plate	Water	-	First electrochemical synthesis of MOFs
Electrochemical technique	Cu plate	Ethanol and water	MTBS electrolyte	
Microwave-assisted technique	Cu(NO_3_)_2_·2H_2_O	Ethanol, DMF, and water	80–100 °C	Comparison with conventional synthesis methods
Mechanochemical technique	Cu(OAC)_2_·H_2_O	-	-	Yields and properties
Sonochemical synthesis method	Cu(OAC)_2_·H_2_O	Ethanol, DMF, and water	-	Time-dependent synthesis

Note: DMF = N,N′-dimethylformamide; MTBS = Tributylmethylammonium methyl sulfate.
